# ROCK1 is a multifunctional factor maintaining the primordial follicle reserve and follicular development in mice

**DOI:** 10.1152/ajpcell.00019.2023

**Published:** 2023-09-04

**Authors:** Tuo Zhang, Huan Lin, Tianhe Ren, Meina He, Wenying Zheng, Yuntong Tong, Bangming Jin, Kaiyun Xie, Ankang Deng, Shiyu Liu, Yuqian Chen, Guoqiang Xu, Tengxiang Chen, Wei Pan, Ziwen Xiao

**Affiliations:** ^1^Prenatal Diagnosis Center in Guizhou Province, The Affiliated Hospital of Guizhou Medical University, Guiyang, People’s Republic of China; ^2^Department of Obstetrics and Gynecology, The Affiliated Hospital of Guizhou Medical University, Guiyang, People’s Republic of China; ^3^Transformation Engineering Research Center of Chronic Disease Diagnosis and Treatment, Department of Physiology, College of Basic Medicine, Guizhou Medical University, Guiyang, People’s Republic of China; ^4^Guizhou Institute of Precision Medicine, Affiliated Hospital of Guizhou Medical University, Guiyang, People’s Republic of China; ^5^Guizhou Provincial Key Laboratory of Pathogenesis & Drug Research on Common Chronic Diseases, Guizhou Medical University, Guiyang, People’s Republic of China; ^6^Department of Cardiovascular Surgery, Xijing Hospital, Fourth Military Medical University, Xi’an, People’s Republic of China

**Keywords:** follicular development, HIPPO, ovary, primordial follicle reserve, ROCK1

## Abstract

The follicle is the basic structural and functional unit of the ovary in female mammals. The excessive depletion of follicles will lead to diminished ovarian reserve or even premature ovarian failure, resulting in diminished ovarian oogenesis and endocrine function. Excessive follicular depletion is mainly due to loss of primordial follicles. Our analysis of published human ovarian single-cell sequencing results by others revealed a significant increase in rho-associated protein kinase 1 (ROCK1) expression during primordial follicle development. However, the role of ROCK1 in primordial follicle development and maintenance is not clear. This study revealed a gradual increase in ROCK1 expression during primordial follicle activation. Inhibition of ROCK1 resulted in reduced primordial follicle activation, decreased follicular reserve, and delayed development of growing follicles. This effect may be achieved through the HIPPO pathway. The present study indicates that ROCK1 is a key molecule for primordial follicular reserve and follicular development.

**NEW & NOTEWORTHY** ROCK1, one of the Rho GTPases, plays an important role in primordial follicle reserve and follicular development. ROCK1 was primarily expressed in the cytoplasm of oocytes and granulosa cell in mice. Inhibition of ROCK1 significantly reduced the primordial follicle reserve and delayed growing follicle development. ROCK1 regulates primordial follicular reserve and follicle development through the HIPPO signaling pathway. These findings shed new lights on the physiology of sustaining female reproduction.

## Graphical abstract

**Figure:**
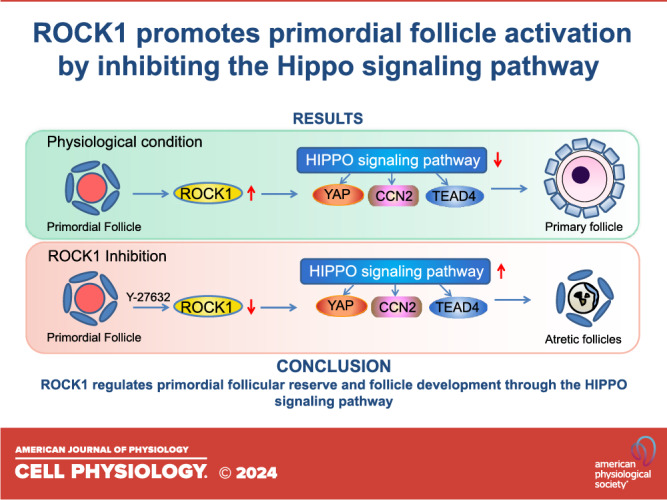

## INTRODUCTION

Diminished ovarian reserve (DOR) implies a quantitative decline in the follicle pool, especially the gradual loss of primordial follicles, which results in a common disease in reproductive-age women during the diagnosis and treatment of infertility (1, 2). Primordial follicles are formed around birth, and all primordial follicles constitute the primordial follicle pool. After primordial follicle formation, the number of follicles does not increase and decreases with age (3). The study of the mechanism of primordial follicle pool maintenance is beneficial for female fertility preservation and treatment of DOR (1, 4).

The primordial follicle consists of the centrally located oocyte and the surrounding pregranulosa cells and their ovarian microenvironment. Oocyte-derived, pregranulosa cell-derived, and microenvironmental changes can lead to abnormal primordial follicle hyperactivation or atresia and lead to reduced ovarian reserve and even premature ovarian failure (5). Key molecules and signaling pathways, such as the TSC1/2-mTOR signaling pathway in pregranulosa cells, TSC1/2-mTOR signaling pathway and PTEN-PI3K-AKT signaling pathway in oocytes, LHX8 and CRL4 complex, have key roles in primordial follicle pool maintenance and primordial follicle activation (6–11). The HIPPO signaling pathway is a serine/threonine kinase signaling cascade that determines organ size and is conserved from Drosophila to mammals (12). HIPPO inhibits the growth of tissues and organs by phosphorylating and inactivating key downstream effector molecules such as YAP, TEAD4, and CCN2 through a kinase cascade reaction. When the HIPPO pathway is inhibited, the tissue organ will overgrow (13–15). Ovarian fragmentation and autotransplantation promote follicle growth by inhibiting the HIPPO pathway (16). Enhancement of actin polymerization is followed by promotion of follicle growth via the HIPPO effector YAP (17). In brief, the HIPPO pathway is important for follicle growth and development. However, the upstream regulatory molecules and mechanisms of the HIPPO pathway during primordial follicle development are not well understood.

Rho-associated protein kinase (ROCK), a downstream effector of Rho GTPases, is a serine-threonine kinase. Mammalian ROCK homologs include ROCK1 and ROCK2, which are key regulators of the actin cytoskeleton and cell polarity (18). Analysis of the single-cell transcriptome sequencing data showed that ROCK1 expression was increased during the development of human ovarian primordial follicles to primary follicles (GSE107746; 19). However, the role of ROCK1 in ovarian follicle development is not clear.

In this study, we investigated whether ROCK1 is necessary for ovarian reserve and follicle development with the help of an ovarian in vitro culture model, a renal dorsal membrane graft model, and an ovarian topical administration model. Inhibition of ROCK1 reduced primordial follicle reserve, delayed primordial follicle activation, and reduced the number of growing follicles. Transcriptome sequencing and experimental verification showed that the HIPPO pathway was activated after inhibition of ROCK1, and further studies by rescue experiments revealed that ROCK1 may regulate follicular reserve and follicle development through the HIPPO pathway. This study may help elucidate the clinical disease of diminished ovarian reserve.

## METHODS

### Animal

Adult CD-1 male and female mice were purchased from Changsha Tianqin Biotechnology Co. and bred to male mice of the same strain. Immunodeficient M-NSG female mice were provided by Shanghai Model Organisms Center, Inc. All mice were housed under controlled lighting (12 h light, 12 h dark) and temperature (24°C–26°C) conditions with free access to food and water. The first 12 h after birth was considered 0 days postpartum (dpp). All procedures were conducted in accordance with the guidelines of and approved by the Animal Research Committee of Guizhou Medical University. All mice were anesthetized by intraperitoneal injection of 1% sodium pentobarbital (50 mg/kg).

### Ovary Organ Culture

An in vitro ovarian culture model was established using standard protocols described previously (20–23). Briefly, the culture of ovarian tissue was mainly divided into two parts: isolation and culture of ovarian tissue. Separating the ovaries involves the following steps: *1*) neonatal 2-day cervical dislocation was performed, and the whole body was decontaminated with 70% alcohol; *2*) ophthalmic forceps were held in the left hand to hold the back half of the mouse; *3*) ophthalmic scissors were held in the right hand to gently cut open the abdomen to expose the internal organs of the mouse, and when the kidney was observed, the internal organs were run toward the head of the mouse, exposing the ovarian tissue located under the kidney; *4*) the carcass of the mouse was cut off by extending the anterior end of the ovary and leaving the ovary in the hindquarters; *5*) the clipped mouse hindquarters was placed in a glass dish containing sterile ice-cold PBS solution, and then, the mouse ovary and ovarian capsule were gently removed with ophthalmic forceps under a microscope and placed in another glass dish containing sterile ice-cold PBS solution; and *6*) the insulin syringe was held with both hands, the ovarian capsule was fixed with the insulin syringe needle with one hand under the microscope, and then, the ovarian capsule was fixed with the insulin syringe needle with the other hand under the microscope. The insulin syringe needle was used to gently scratch the ovarian capsule and expose the ovary. Special care is needed to ensure that the ovary is not damaged during the entire process of separating the ovary. Second, the separation of ovarian tissue consists of the following steps: *1*) 1.2 mL of Dulbecco’s modified Eagle’s medium/Ham F12 nutrient mixture (Gibco, Life Technologies, CA) was added with insulin-transferrin-sodium selenite (Sigma, 1:100) and penicillin-streptomycin solution to each well of a six-well plate with low adsorption, and the number of wells was determined according to the experimental group. The medium-containing six-well plate was equilibrated in an incubator for 10–15 min. *2*) Ovaries were transferred to the six-well plate for incubation under a microscope using a 50-μL pipette, and 6–10 ovaries were placed in each well. The tip of the gun was blunted to prevent the tip from damaging the ovarian tissue. *3*) The medium was changed every other day to ensure that the ovaries were in suspension throughout the culture process. If the ovaries sink or float to the surface of the petri dish, the blunt tip can be used to gently move the position of the ovaries to keep them in suspension. *4*) Ovaries were randomly distributed to the control and treated groups. The ovaries were incubated with 10 μM Y-27632 (SELLECK, S6390) and 500 nM YAP-TEAD Inhibitor 1 (SELLECK, S8164). Ovaries were collected at 48 and 72 h.

### Kidney Capsule Transplantation

All surgical instruments used during the experimental process were sterilized by autoclaving to prevent surgical infections from affecting the experimental results. In particular, immunodeficient mice need to be carefully operated on. *1*) Healthy immunodeficient female mice at 6–8 wk of age were selected as recipients for ovarian renal peritoneal transplantation. *2*) The mice were weighed and injected intraperitoneally with 0.15 mL/10 g of tribromoethanol solution and were generally anesthetized 1–2 min after the injection. At this time, the mice were gently pinched with tweezers on the toes, and the mice usually did not resist violently; it was normal if they resisted slightly, which did not affect the surgical operation. At this time, the eyes, paws, and tails of the mice were full of blood. The next surgical operation was carried out after the anesthesia was working; otherwise, the anesthetics were appropriately increased. *3*) The hair on the back of the mice was trimmed cleanly with electric hair-cutting scissors, and the skin on the back of the mice was fully and thoroughly sterilized with 75% alcohol to prevent surgical infection. *4*) With sterile ophthalmic scissors, the skin was cut along the spine from the caudal to cephalic direction at the approximate location of the dorsal kidney of the mice, with an incision of ∼1.5 cm, taking care to avoid blood vessels. *5*) The skin was bluntly separated from the peritoneum with forceps, taking care not to damage blood vessels. *6*) A 1-cm incision was made in the peritoneum directly above the kidney on the side of the implanted ovary, taking care to avoid blood vessels. Blood vessels were carefully avoided. *7*) Using a syringe needle, we gently made a small lateral incision in the renal peritoneum, which is slightly smaller than the size of the implanted ovarian tissue. We used a curved blunt glass tube to open the renal peritoneum, pipette the ovary into the incision, and then gently move the implanted ovary away from the opening of the renal peritoneum as far as possible with the curved blunt glass tube to prevent the implanted ovary from slipping out of the peritoneal peritoneum. The ovaries of the control and treatment groups were transplanted under the renal peritoneum on both sides of the same recipient rats, as shown in Fig. 2*F*. *8*) The fat pads of the ovaries were held in place with forceps. The ovaries were removed on both sides by gently tipping the union of the ovary and oviduct with burnt ophthalmic scissors and treated with antibiotics. *9*) The kidneys were gently returned to their original position, the peritoneal openings were sutured, the openings in the skin were sutured, and antibiotics and iodine were applied to the wounds to prevent infection. *10*) The mice were placed in a warm environment to wake up, and after awakening, we observed the mental status of the mice and the subsequent recovery of the wounds. After surgery, mice tend to be thirsty, so care should be taken to supply water and food in a timely manner. *11*) Two weeks after transplantation, mice were killed by cervical dislocation, and the kidneys were removed. The mouse kidney peritoneum was gently opened, and the ovarian tissue implanted under the peritoneum was carefully collected for subsequent experimental studies.

### Ovarian Topical Administration In Vivo

Typical ovarian administration was performed according to the previous report (21, 22). Briefly, 3-wk-old CD-1 female mice were anesthetized with avertin (300 mg/kg, T48402, Sigma), and the ovaries were gently exposed from the incisions on the backs, as illustrated in Supplemental Fig. 4*A*. Y-27632 (100 μM) was dissolved in liquid growth factor-reduced matrigel (354230, BD) on ice. In the control group, 10 μL of precooled growth factor-reduced matrigel (354230, BD) was injected into the unilateral ovarian bursa using an insulin syringe under microscope (S9E, Leica, Germany). The contralateral ovary was injected with 10 μL of matrigel containing Y-27632. After the temperature-sensitive matrigel solidified, the incisions were sutured. Ovarian tissues were collected 14 days after surgery.

### Ovarian Section Immunofluorescence

Fresh ovaries were fixed overnight in 4% paraformaldehyde, dehydrated in gradient alcohol, cleared in xylene, embedded in paraffin, and sectioned at 5 μm. Ovarian sections were deparaffinized, rehydrated, and subjected to high-pressure antigen repair with 0.01% sodium citrate buffer for 20 min. The sections were then rinsed thoroughly with PBS for 10 min, blocked with 5% BSA in PBS for 1 h at room temperature, and incubated with primary antibodies overnight at 4°C. The primary antibodies used are listed in Supplemental Table S1. Next, ovarian sections were rinsed thoroughly with PBS for 1 h and incubated with Alexa Fluor 488- or 555-conjugated secondary antibody (1:100; Beyotime Biotechnology, PR China) for 1 h at 37°C. The ovarian sections were rinsed thoroughly with PBS, stained with DAPI (1:100; C1011, Beyotime Biotechnology, PR China) for 5 min, and sealed in antifade fluorescence mounting medium (C1210, Applygen, PR China) with coverslips. Sections were examined and photographed using an Olympus laser scanning confocal microscope (U-TBI90, Japan) in a dark environment at 22°C, and the objective lens was ×20. All images were obtained by Olympus OlyVIA software combined with Adobe Illustrator CC 2019. All the pictures of the control and treatment groups were not altered for intensity and/or contrast.

### TUNEL Assay

The TUNEL assay was performed with ovarian paraffin sections, according to the instructions provided with the One Step TUNEL Apoptosis Assay Kit (C1086, Beyotime, PR China). Sections were examined and photographed using Olympus OlyVIA.

### Quantification of Ovarian Follicles

The whole ovarian follicle count was determined according to the well-accepted standards. Serial sections of each ovary (8-μm thick) were stained with hematoxylin. All serial sections of each ovary were counted. Ovarian follicles at different developmental stages, including primordial follicles (PmF), primary follicles (PF), secondary follicles (SF), and antral follicles (AF), were counted in collected sections of an ovary. Only follicles containing clearly visible nuclei of oocytes in each individual section were counted.

### Western Blotting Analysis

The ovarian protein samples were extracted in WIP tissue lysis buffer containing phenylmethylsulfonyl fluoride (8553S, Cell Signaling Technologies), according to the manufacturer’s instructions. The protein concentration was measured by a bicinchoninic acid (BCA) assay (P0012, Beyotime, PR China). The samples were separated by 10%–15% SDS-PAGE and then transferred to polyvinylidene fluoride membranes (IPVH00010, Millipore). The membranes were blocked with 5% skim milk powder for 2 h at room temperature and then incubated with appropriate primary antibodies overnight at 4°C. The primary antibodies used are listed in Supplemental Table S1. The membranes were rinsed thoroughly with Tris-buffered saline-Tween 20 (TBST) for 30 min. The membranes were incubated with the secondary antibody for 1 h at room temperature and rinsed thoroughly with TBST for 30 min. The secondary antibodies were horseradish peroxidase-conjugated goat anti-mouse IgG (H + L) (ZB-2305, Zhongshan Golden Bridge, PR China) and horseradish peroxidase-conjugated goat anti-rabbit IgG (H + L) (ZB-5301, Zhongshan Golden Bridge, PR China) diluted 1:10,000. The membranes were visualized using a SuperSignal West Pico Chemiluminescent Detection System (5200, Tanon, PR China). β-actin was used as the intrinsic control.

### Real-Time PCR Analysis

Total RNA was extracted with TRIzol reagent (Invitrogen, Carlsbad, CA), according to the manufacturer’s protocol. cDNA was synthesized by reverse transcription using 1–3 µg of total RNA. RT-PCR, which is semiquantitative, was performed using SYBR Select Master Mix and a Bio-Rad CFX96 Real-Time PCR system. The data were normalized to those of β-actin. The primers used are listed in Supplemental Table S1.

### RNA-Seq and Data Analysis

The 2 dpp ovaries cultured with Y-27632 for 2 days were collected to extract total RNA as described earlier. RNA-seq was performed by LC-Bio Technology Co., Ltd. (Hangzhou, PR China). Differentially expressed genes (DEGs) were identified between the two groups at a *P* value of <5% and absolute log2FoldChange ≥ 1. The differentially expressed genes are shown in Supplemental Table S2. For determination of the function of the differentially expressed genes, gene ontology (GO) enrichment and Kyoto encyclopedia of genes and genomes (KEGG) pathway enrichment analyses were performed. Bioinformatic analysis was performed using the OmicStudio tools at https://www.omicstudio.cn/tool. Gene set enrichment analysis (GSEA) was performed using GSEA software.

### Statistical Analyses

Statistical analyses were carried out using GraphPad Prism 8.0. Data are expressed as the mean of at least three independent experiments. The results are given as the means ± SD. Two-tailed unpaired Student’s *t* test and one-way analysis of variance followed by Tukey’s post hoc test were used to analyze the statistical significance between two groups and among multiple groups, respectively. The statistical significance was set at *P* value < 0.05.

## RESULTS

### Expression of ROCK1 Increases during Primordial Follicle Activation

**Figure 1.**
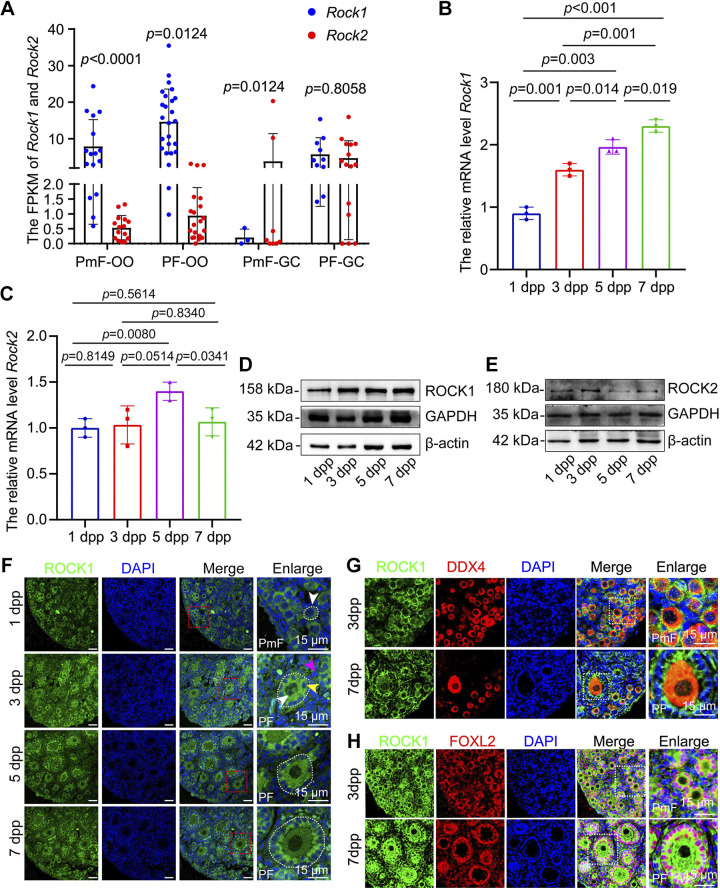
ROCK1 expression was increased during primordial follicle activation. *A*: single-cell sequencing results (GSE107746) were analyzed for changes in *Rock1* and *Rock2* expression in granulosa cells and oocytes of human primordial and primary follicles. *B* and *C*: the total mRNA levels of *Rock1* and *Rock2* in female mouse ovaries from 1 to 7 dpp (*n* = 3). *D* and *E*: the total protein levels of ROCK1 and ROCK2 in female mouse ovaries from 1 to 7 dpp. Every group included 10 ovaries, and the experiment was repeated at least three times. *F*: immunofluorescent staining of ROCK1 in newborn mice ovaries. ROCK1 signals (green) were costained with DDX4 (red). The nuclei were stained with DAPI (blue). White arrows indicate oocytes, yellow arrows indicate granulosa cells, and purple arrows indicate interstitial cells. Scale bar: 15 μm. *G*: immunofluorescent staining of ROCK1 in newborn mice ovaries. ROCK1 signals (green) were costained with DDX4 (red). The nucleus was stained by DAPI (blue). Scale bar: 15 μm. *H*: immunofluorescent staining of ROCK1 in newborn mice ovaries. ROCK1 signals (green) were costained with FOXL2 (red). The nuclei were stained with DAPI (blue). Scale bar: 15 μm. Statistical significance was determined using two-tailed unpaired Student’s *t* test (*A*) or one-way ANOVA followed by Tukey’s post hoc test (*B* and *C*), and values are the means ± SD. Statistically significant values of *P* < 0.05. PF, primary follicle; PmF, primordial follicle; ROCK1, rho-associated protein kinase 1; 2 dpp, 2 days postpartum.

To investigate the potential role of ROCK1 in early follicular development, we first analyzed published single-cell transcriptome sequencing data from human follicular granulosa cells and oocytes at different developmental stages. Zhang et al. collected follicles at different developmental stages from adult ovaries and performed single-cell transcriptome sequencing of oocytes and granulosa cells. We used data from this database (GSE107746) to analyze the expression of *Rock1* and *Rock2* in granulosa cells and oocytes during the development of primordial follicles to primary follicles. The results showed that ROCK1 expression was significantly higher than ROCK2 expression in primordial and primary follicles. ROCK1 expression was higher in primary follicles than in primordial follicles in both granulosa cells and oocytes (Fig. 1*A*). We further used 1-, 3-, 5-, and 7-dpp mouse ovaries to detect the expression of ROCK1 and ROCK2 during primordial follicle pool establishment and primordial follicle activation. The results showed that the change in ROCK2 expression from 1 to 7 dpp was not significant, and the expression of ROCK1 increased significantly (Fig. 1, *B* and *C*). The results of Western blotting showed that the expression of ROCK1 was increased from 1 to 7 dpp. However, the expression of ROCK2 was unchanged (Fig. 1, *D* and *E*). The immunofluorescence results showed that ROCK1 was expressed in the cytoplasm of both oocytes and somatic cells, including pregranulosa cells, granulosa cells, and interstitial cells from primordial follicles and primary follicles (Fig. 1, *F*–*H*). This result is basically consistent with the changes in human ovaries. Given the expression pattern of ROCK1 in primordial follicle pool establishment and primordial follicle activation, the role of ROCK1 in early follicle development should be explored.

### ROCK1 Is Indispensable for Maintaining the Primordial Follicle Reserve

**Figure 2.**
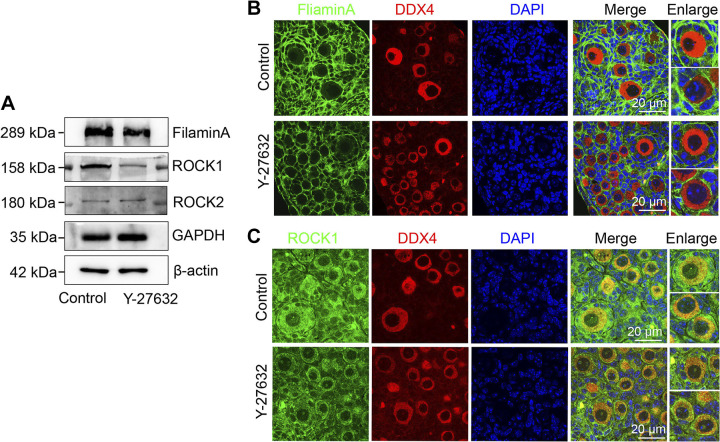
The efficacy of Y-27632 inhibitor. *A*: the protein expression levels of FilaminA, ROCK1, and ROCK2. The 2 dpp ovaries were cultured with or without Y-27632 for 2 days. Each group was 10 ovaries, repeat for at least three times. GAPDH and β-actin served as an internal control. *B*: immunofluorescence of FilaminA within Y-27632-treated ovaries. The 2 dpp ovaries were cultured with or without Y-27632 for 2 days. ROCK1 signals (green) were costained with DDX4 (red). The nucleus was stained by DAPI (blue). Scale bar: 20 μm. *C*: immunofluorescence of ROCK1 within Y-27632-treated ovaries. The 2 dpp ovaries were cultured with or without Y-27632 for 2 days. ROCK1 signals (green) were costained with DDX4 (red). The nucleus was stained by DAPI (blue). Scale bar: 20 μm. ROCK1, rho-associated protein kinase 1; 2 dpp, 2 days postpartum.

**Figure 3.**
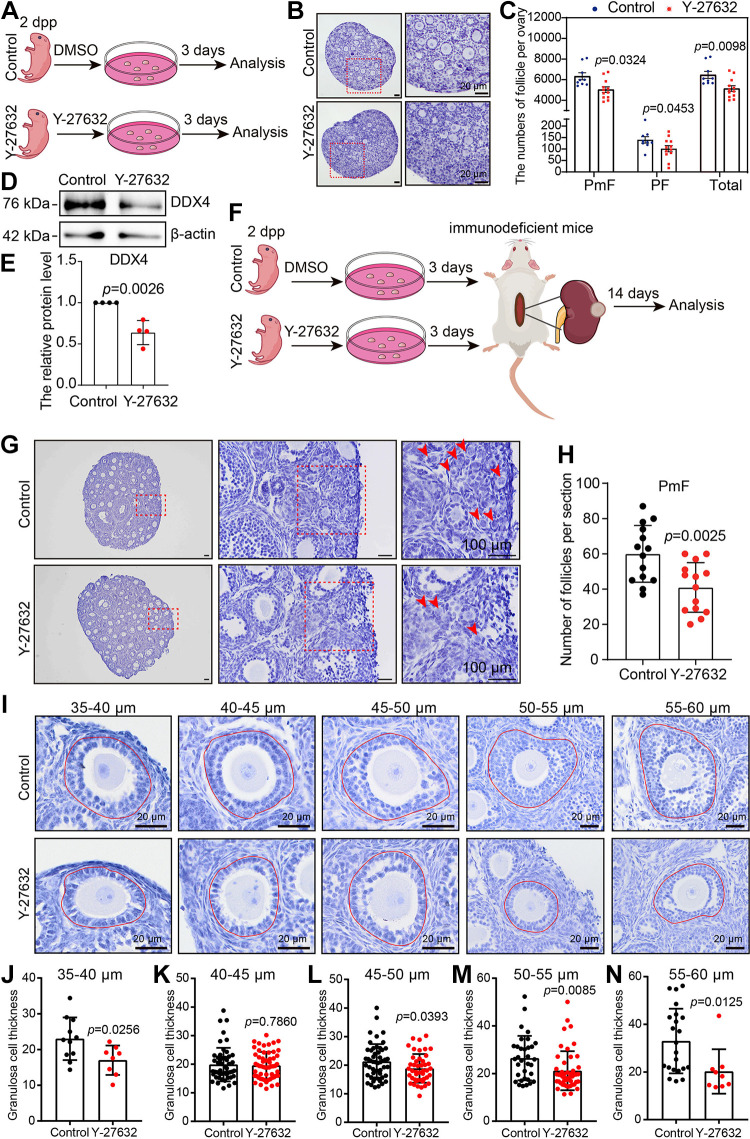
ROCK1 regulates follicle activation, maintenance, and development. *A*: experimental design for culture of the newborn female mouse ovaries from 2 dpp mice with the ROCK1 inhibitor Y-27632 at 10 μM (*n* = 9). *B* and *C*: hematoxylin staining and whole ovary follicle counting of ovarian sections cultured for 3 days with Y-27632. Scale bar: 20 μm (*n* = 9). *D*: Western blot to detect changes in DDX4. The 2 dpp female ovaries were cultured for 2 days. Each group included 10 ovaries, and the experiment was repeated at least three times. *E*: results of the statistical analysis of *D* (*n* = 4). *F*: experimental design for kidney capsule transplantation of Y-27632-treated ovaries. *G*: hematoxylin staining of ovarian sections treated with Y-27632. Female mouse ovaries at 3 dpp were cultured with Y-27632 for 3 days and transplanted into immunodeficient mice to continue development under the kidney capsule for 14 days. Scale bar: 20 μm. *H*: primordial follicle (PmF) counting of ovarian sections from kidney capsule transplantation (*n* = 14). *I*: Hematoxylin eosin (HE) staining of ovarian sections from kidney capsule transplantation. Scale bar: 20 μm. *J*: quantitation of granulosa cell thickness from oocytes diameters of 35–40 μm. *n* = 8. *K*: quantitation of granulosa cell thickness from oocytes diameters of 40–45 μm (*n* = 46). *L*: quantitation of granulosa cell thickness from oocytes diameters of 45–50 μm (*n* = 42). *M*: quantitation of granulosa cell thickness from oocytes diameters of 50–55 μm (*n* = 32). *N*: quantitation of granulosa cell thickness from oocytes diameters of 55–60 μm (*n* = 9). Statistical significance was determined using two-tailed unpaired Student’s *t* test, and values are the means ± SD. Statistically significant values are indicated by *P* < 0.05. ROCK1, rho-associated protein kinase 1; 2 dpp, 2 days postpartum.

**Figure 4.**
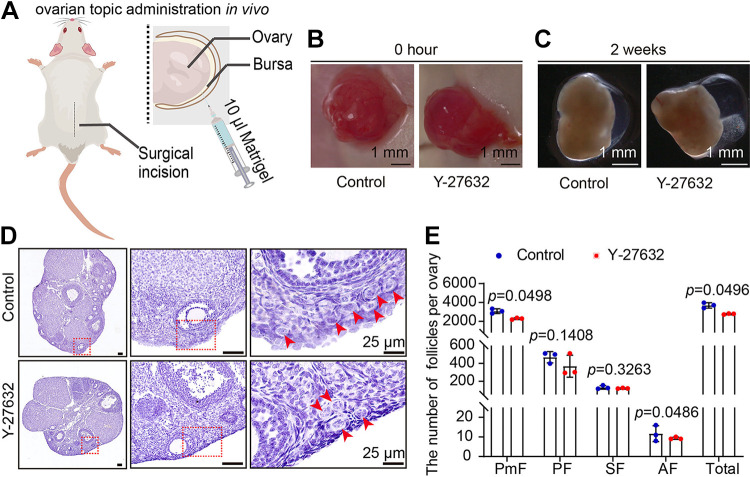
ROCK1 inhibition leads to reduced primordial follicles and antral follicles in ovarian topical administration model. *A*: experimental design for ovarian topical administration in vivo. *B*: photograph of the ovary at 0 h after matrigel injection. The same female mice were injected with matrigel containing Y-27632 on one side of the ovary and blank matrigel on the other side. *C*: photograph of the ovary at 2 wk after matrigel injection. *D* and *E*: hematoxylin staining and whole ovary follicle counting of ovarian sections after ovarian topical administration for 14 days with Y-27632 (*n* = 3). The results for two experimental groups were compared by two-tailed unpaired Student’s *t* tests. Statistical significance was determined using two-tailed unpaired Student’s *t* test, and values are the means ± SD. Statistically significant values are indicated by *P* < 0.05. ROCK1, rho-associated protein kinase 1.

To investigate the role of ROCK1 in primordial follicle development, we utilized an established ovarian in vitro culture model (8, 20, 21). To test the inhibitory effect of Y-27632 inhibitor, after adding Y-27632 for ovarian culture, we detected the changes in the protein levels of ROCK1, ROCK2, and FilaminA, the downstream molecule of ROCK1. The results showed that ROCK1 and Filamin A protein levels decreased significantly after 2 days of culture, whereas ROCK2 protein levels did not change significantly (Fig. 2*A*). The results of immunofluorescence showed that the fluorescence intensity of FilaminA and ROCK1 was significantly reduced in the Y-27632 group compared with the control group (Fig. 2, *B* and *C*). These results indicate that ROCK1 was successfully inhibited. At 2 dpp, ovaries were exposed to Y-27632 (ROCK1 inhibiting group) or DMSO (control group) for 3 days before analysis (Fig. 3*A*), and the changes in the number of primordial and primary follicles were observed and counted. The results showed that the numbers of primordial follicles, primary follicles, and total follicles decreased significantly (Fig. 3, *B* and *C*). Western blotting showed reduced expression of the oocyte marker DDX4 (Fig. 3, *D* and *E*). To observe follicle development after Y-27632 treatment for a longer period of time, we transplanted ovaries incubated with Y-27632 for 3 days into immunodeficient mice under the kidney capsule and continued to grow for 2 wk (Fig. 3*F*). The results showed a significant reduction in the number of primordial follicles and a delay in the development of growing follicles (Fig. 3, *G* and *H*). Granulosa cell thickness was significantly reduced for the same oocyte diameter (Fig. 3, *I*–*N*). We further investigated the effect of ROCK1 on follicular development using a model of ovarian topical administration in vivo (21, 22). The ovaries of 3-wk-old mice were given local in vivo injections of matrigel containing Y-27632, and ovaries were collected 2 wk after injection (Fig. 4, *A*–*C*). The results showed a significant decrease in the number of primordial follicles, antral follicles, and total follicles compared with those of the control group (Fig. 4, *D* and *E*). The results of all three models show that inhibition of ROCK1 leads to a reduction in ovarian reserve, especially in the number of primordial follicles, which is necessary for the maintenance of follicular reserve.

### Inhibition of ROCK1 Leads to Decreased Proliferation and Increased Apoptosis in Ovarian Cells

**Figure 5.**
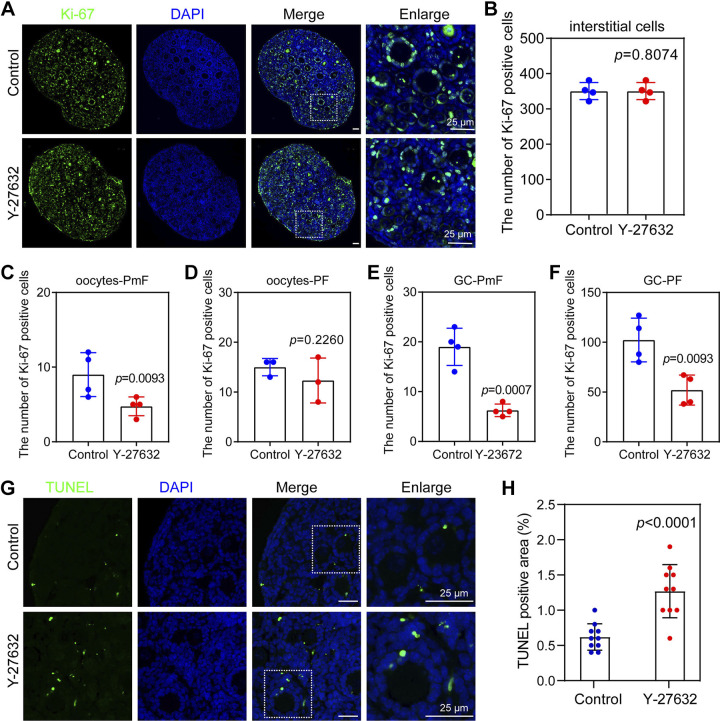
Y-27632-treated ovaries showed reduced proliferation and increased apoptosis. *A*: immunofluorescence of *K*_i_-67 in Y-27632-treated ovaries. ROCK1 signals (green) were costained with DAPI (blue). The 2 dpp ovaries were cultured with or without Y-27632 for 2 days. Scale bar: 25 μm. *B*: quantitation of *K*_i_-67-positive cells from interstitial cells (*n* = 4). *C*: quantitation of *K*_i_-67-positive cell from oocytes of primordial follicle (oocytes-PmF) (*n* = 4). *D*: quantitation of *K*_i_-67-positive cells from oocytes of primary follicle (oocytes-PF) (*n* = 4). *E*: quantitation of *K*_i_-67-positive cells from pregranulosa cell of primordial follicle (GC-PmF) (*n* = 4). *F*: quantitation of *K*_i_-67-positive cells from granulosa cell of primordial follicle (GC-PF) (*n* = 4). *G*: immunofluorescence of TUNEL with Y-27632-treated ovary. Scale bar: 25 μm. *H*: quantitation of TUNEL-positive cell from whole ovary (*n* = 10). Statistical significance was determined using two-tailed unpaired Student’s *t* test, and values are the means ± SD. Statistically significant values of *P* < 0.05. 2 dpp, 2 days postpartum.

To observe the proliferation and apoptosis of ovaries after ROCK1 inhibition, we first performed immunofluorescence staining of *K*_i_-67 on ovarian slides and counted the number of *K*_i_-67-positive cells (Fig. 5, *A*–*F*). The results showed a significant decrease in the number of *K*_i_-67-positive cells in interstitial cells (Fig. 5*B*), oocytes of primordial follicles (Fig. 5*C*), oocytes of primary follicles (Fig. 5*D*), pregranulosa cells of primordial follicles (Fig. 5*E*), and granulosa cells of primary follicles (Fig. 5*F*). TUNEL staining showed a significant increase in the number of TUNEL-positive cells in the Y-27632 group compared with the control group (Fig. 5, *G* and *H*). These results suggest that inhibition of ROCK1 leads to reduced proliferation and increased apoptosis in the ovary.

### ROCK1 May Regulate the Follicular Reserve through the HIPPO Pathway

**Figure 6.**
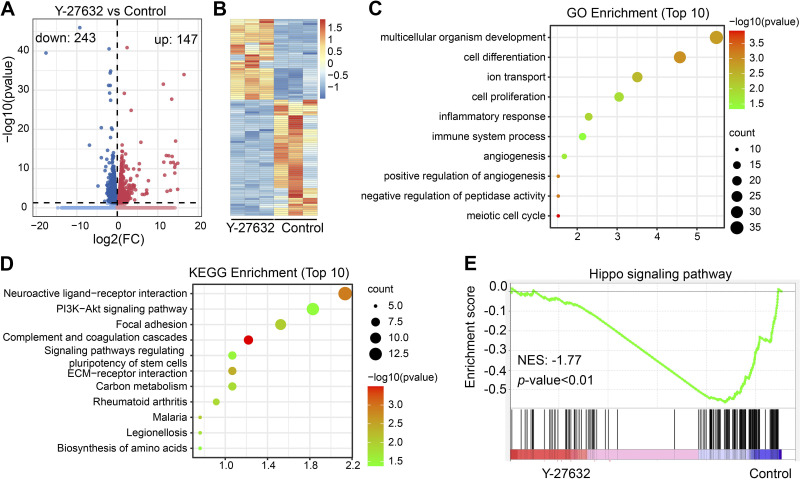
Transcriptome sequencing results indicate that ROCK1 may regulate follicular reserve through the HIPPO pathway. *A*: volcano plots show the changes in mRNA abundance in RNA-seq of Y-27632-treated ovary. DEGs were identified from Y-27632 vs. the control (*n* = 3). *B*: heatmap of significantly altered genes. *C*–*E*: representative enriched GO terms, KEGG terms, and GSEA term. DEGs, differentially expressed genes; GO, gene ontology; KEGG, Kyoto encyclopedia of genes and genomes.

**Figure 7.**
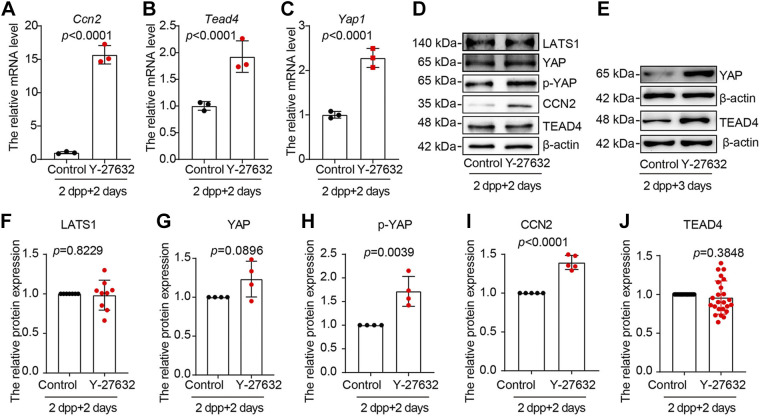
Y-27632-treated ovaries showed enhancement of HIPPO signaling pathway. *A*–*C*: the mRNA expression levels of *Ccn2*, *Tead4*, and *Yap1*. The 2 dpp ovaries were cultured with or without Y-27632 for 2 days (*n* > 3/group). *D*: the protein expression levels of LAST1, YAP, p-YAP, CCN2, and TEAD4. The 2 dpp ovaries were cultured with or without Y-27632 for 2 days. Each group included 10 ovaries, and the experiment was repeated at least three times. *E*: the protein expression levels of YAP and TEAD4. The 2 dpp ovaries were cultured with or without Y-27632 for 3 days. Each group included 10 ovaries, and the experiment was repeated at least three times. *F*–*J*: results of the statistical analysis of *D*. The results are given as the means ± SD. The results for two experimental groups were compared by two-tailed unpaired Student’s *t* tests. Statistical significance was determined using two-tailed unpaired Student’s *t* test, and values are the means ± SD. Statistically significant values are indicated by *P* < 0.05. 2 dpp, 2 days postpartum.

**Figure 8.**
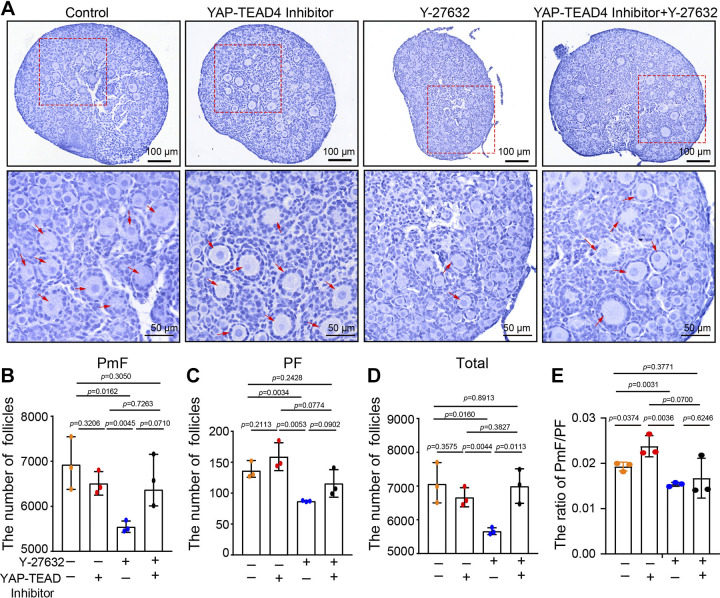
ROCK1 regulate follicle reserve through HIPPO signaling pathway. *A*: hematoxylin staining of ovarian sections from Y-27632 and YAP-TEAD4 inhibitor-treated female ovaries. The 2 dpp ovaries were cultured for 3 days. *B*–*D*: whole ovary follicle counting. *E*: the ratio of PmF/PF (*n* = 3). Statistical significance was determined using one-way ANOVA followed by Tukey’s post hoc test, and values are the means ± SD. The results are given as the means ± SE. The results for two experimental groups were compared by two-tailed unpaired Student’s *t* tests. Statistical significance was determined using two-tailed unpaired Student’s *t* test, and values are the means ± SD. Statistically significant values are indicated by *P* < 0.05. PF, primary follicle; PmF, primordial follicle; total, total follicle; 2 dpp, 2 days postpartum.

To investigate how ROCK1 regulates the follicular reserve, we collected Y-27632-cultured ovaries for transcriptome sequencing, and transcriptome analysis identified 243 downregulated genes and 147 upregulated genes (Fig. 6, *A* and *B*). GO enrichment analysis of differentially expressed genes revealed significant enrichment during biological processes such as organ growth, cell differentiation, iNO transport, and cell proliferation (Fig. 6*C*). KEGG pathway enrichment analysis showed enrichment of signaling pathway bundles such as neuroactive ligand-receptor interaction, PI3K-AKT, signaling pathway, and focal adhesion (Fig. 6*D*). Gene set enrichment analysis (GSEA) is a computational method that determines whether an a priori defined set of genes shows statistically significant, concordant differences between two biological states (e.g., phenotypes). The normalized enrichment score (NES), nominal *P* value, and false-discovery rate (FDR) *q* value indicated the importance of the association between gene sets and pathways. Only gene sets with |NES| > 1, *P* value < 0.05, and *q* value < 0.25 were considered significant. Only several leading gene sets are displayed in the plot. GSEA showed significant enrichment in the HIPPO signaling pathway (Fig. 6*E*). Studies have shown that the HIPPO signaling pathway is essential for primordial follicle activation, maintenance of primordial follicle reserve, and growing follicle development (16, 17, 23–26). We examined key effector molecules in the HIPPO signaling pathway, and the results showed that the mRNA levels of *Ccn2*, *Tead4*, and *Yap1* were significantly increased (Fig. 7, *A*–*C*). Western blot results showed that the protein levels of CCN2, p-YAP, and YAP were significantly increased, and the protein level of TEAD4 was unchanged (Fig. 7, *D*–*J*). However, when the in vitro culture time was extended to 3 days, the protein levels of YAP and CCN2 increased significantly in the Y-27632 group (Fig. 7*E*). These results suggest that ROCK1 may maintain primordial follicular reserve in mice through the HIPPO pathway. To prove this hypothesis, we inhibited ROCK1 along with the HIPPO pathway. Histological observations and whole ovary follicular count statistics showed that inhibition of both ROCK1 and HIPPO pathways reversed the reduction in follicular reserve caused by ROCK1 inhibition (Fig. 8, *A*–*E*). These results suggest that ROCK1 regulates primordial follicular reserve in mice via the HIPPO pathway.

## DISCUSSION

The follicle is a nonrenewable reproductive resource for females (3). Loss of follicles will lead to premature aging of the ovaries and clinical disorders such as endocrine disorders and osteoporosis. Loss of the primordial follicle, which is the starting point of follicular development after birth, will directly lead to a reduction in follicular reserve (27). In this study, we found that ROCK1 is essential for primordial follicular reserve maintenance and follicular development, and this function may be regulated through the HIPPO pathway.

Since birth, the number of follicles in the ovary does not increase. After the number of primordial follicles falls below 1,000, women will face menopause. An abnormal decrease in the primordial follicle reserve will lead to premature aging of the ovaries. Most immature oocytes remain dormant in primordial follicles in the ovary. Most of the primordial follicles in the ovary are dormant to ensure the reproductive lifespan of the female (5). The PI3K and mTOR signaling pathways were found to be essential for primordial follicle activation and follicular reserve maintenance (9, 28–30). We found that inhibition of ROCK1 did not affect the protein levels and phosphorylation levels of key molecules of the PI3K and mTOR signaling pathways (data not shown). ROCK1 may not regulate primordial follicle activation and reserve maintenance through the PI3K and mTOR signaling pathways. We found that inhibition of ROCK1 was followed by activation of the HIPPO pathway, leading to a reduction in primordial follicle activation and primordial follicular reserve. Reduced follicle stocks and impaired development of growing follicles were observed in ROCK1 inhibitor-treated mouse ovaries by renal dorsal membrane transplantation. These results suggest that ROCK1 is necessary for follicle development and may play a critical role in the maintenance of the female reproductive lifespan. These results suggest that ROCK1 is required for follicle development and may play a critical role in the maintenance of female reproductive lifespan. However, further studies using transgenic mouse models are needed to fully demonstrate the role of ROCK1 in reproductive lifespan.

Rho family mechano-signaling through the actin cytoskeleton positively regulates physiological TEAD/YAP transcription (31). HIPPO signaling pathway-related molecules in the ovary were found to be necessary for oocyte survival, follicle development, granulosa cell fate maintenance, and embryo development (16, 32, 33). YAP1 encodes a downstream nuclear effector of the Hippo signaling pathway (34, 35). Knockdown of *Yap1* in granulosa cells with *Foxl2*-Cre resulted in increased apoptosis of granulosa cells, a decreased number of corpus lutea, smaller ovaries, and subfertility in female mice. However, knockdown of *Yap1* in luteinized granulosa cells with *Cyp19*-Cre had no effect on ovarian morphology, and female mice were fertile (32). Interestingly, knockdown of *Last1* and *Last2*, negative regulators of YAP1 in the HIPPO signaling pathway, in luteal granulosa cells with *Cyp19*-Cre resulted in transdifferentiation of ovarian granulosa cells into multiple lineages, including Sertoli-like cells and osteoblasts, and female mice were subfertile (33). Oocyte expression of YAP1 is nonessential for follicular development but is a key activator of the early zygotic genome (36, 37). However, less is known about the HIPPO pathway in early follicular development, especially in primordial follicles. Our study shows that the HIPPO pathway is necessary for the maintenance and activation of primordial follicle reserve. ROCK1 may be the upstream regulatory signal of HIPPO during the early development of primordial follicles. However, limited by the ovarian culture model in vitro, it is not clear in which cells this role is exerted, such as primordial follicle oocytes, pregranulosa cells, and interstitial cells. How ROCK1 regulates the HIPPO pathway needs to be further investigated in the future. Studies have shown that mechanical stress is involved in primordial follicle activation (38). YAP/TAZ are major mediators of mechanical stress signals, and F-actin levels can be influenced by the mechanical environment through tension sensing at adherens junctions, focal adhesions, and nuclei (39). ROCK1, as a small GTPase, regulates actin cytoskeleton reorganization processes and the resulting overall behavior of cells (40, 41). ROCK1 and HIPPO are important mechanical pressure-sensing molecules, and whether they may regulate follicle development through mechanical pressure alteration may be worth investigating in the future.

## ETHICAL APPROVALS

All animal procedures were conducted in accordance with the guidelines of and approved by the Animal Research Committee of the Guizhou Medical University.

## DATA AVAILABILITY

All data generated or analyzed during this study are included in this published article and its supplementary information files. Any additional information required to reanalyze the data reported in this paper is available from the lead contact upon request.

## SUPPLEMENTAL DATA

10.6084/m9.figshare.23731494.v1 Supplemental materials: https://doi.org/10.6084/m9.figshare.23731494.v1.

## GRANTS

This study is funded by the National Natural Science Foundation of China (32100686 to T. Zhang, 32100913 to M. He, and 82260291 to T. Zhang), Guizhou Provincial Science and Technology Projects (ZK[2023]314 to T. Zhang and ZK(2022)346 to M. He), Science and Technology Fund Project of Guizhou Provincial Health Commission (gzwkj2021-299 to T. Zhang and gzwkj2021-527 to M. He), China Postdoctoral Science Foundation (2022M710919 to T. Zhang and 2021M700972 to M. He), Excellent Young Talents Plan of Guizhou Medical University ([2022]110 to T. Zhang and [2023]111 to M. He), Doctoral Startup Foundation of Guizhou Medical University ((2020)038 to T. Zhang and (2020)039 to M. He), the National Natural Science Foundation Cultivation Project of Guizhou Medical University (20NSP031 to T. Zhang and 20NSP008 to M. He), the Continuous Support Fund for Excellent Scientific Research Platform of Colleges and Universities in Guizhou Province (QJJ (2022) 020 to T. Chen), Guizhou Provincial Natural Science Foundation (Grant Nos. (2021)4029 and (2022)4017), and Innovation and Entrepreneurship Training Program for College Students in Guizhou Province (202110660058, S202210660136, S202210660093, 202210660090, 202110660058).

## DISCLOSURES

No conflicts of interest, financial or otherwise, are declared by the authors. 

## AUTHOR CONTRIBUTIONS

T.Z. conceived and designed research; T.Z., H.L., T.R., W.Z., Y.T., K.X., A.D., S.L., and Y.C., performed experiments; T.Z., H.L., M.H., W.Z., B.J., G.X., T.C., W.P., and Z.X. analyzed data; T.Z., M.H., G.X., T.C., W.P., and Z.X. interpreted results of experiments; T.Z., H.L., G.X., T.C., and Z.X. prepared figures; T.Z., M.H., G.X., T.C., W.P., and Z.X. drafted manuscript; T.Z., W.P., and Z.X. edited and revised manuscript; T.Z., H.L., T.R., M.H., W.Z., Y.T., B.J., G.X., T.C., W.P., and Z.X. approved final version of manuscript. 
